# A Simple and Easy Intramedullary Lavage Method to Prevent Embolism During and After Reamed Long Bone Nailing

**DOI:** 10.7759/cureus.1609

**Published:** 2017-08-25

**Authors:** MN Baig, W Curtin

**Affiliations:** 1 Orthopaedics, Galway University Hospital

**Keywords:** fat embolism

## Abstract

Reaming of the long bones is widely practiced because it allows for improved healing and early mobilization in patients needing surgical debridement of bone tissue. The insertion of reamed intramedullary nails can cause complications such as bone necrosis, cortical blood supply damage, and fat or bone marrow embolism. We describe a novel way to limit the amount of material in the canal before nail insertion to limit the chances of embolism.

## Introduction

The intramedullary (IM) nailing of the long bones was introduced by Kuntscher in the mid-twentieth century. Its popularity and success have increased over time with improvements in understanding and advances in implants. Currently, it is widely used because it allows for good control over the alignment and rotation of the long bones [1]. To ream or not is debated, but the evidence available in the literature inclines toward reaming.

Evidence reported in favor of IM reaming include the use of stronger/wider diameter nails, better biomechanical stability, intact periosteal vasculature, and the osteogenic effect of reaming debris. The disadvantages attributed to reaming include heat necrosis, fat/bone marrow embolism, and cortical blood supply damage [2].

Among these, fat/bone marrow embolism can progress to acute respiratory distress syndrome (ARDS) via a condition known as fat embolism syndrome (FES). While some surgeons advocate various techniques to vent the IM canal to decrease the chances of embolism [3], this technical report describes our technique.

## Technical report

We have practiced our new technique to minimize the risk of fat embolism during and after reaming. Our technique requires no specific or customized instruments, and it is based on the standard IM nailing procedure instrumentation.

In standard IM nailing, an incision entry point for femoral or tibial nailing is made, followed by the use of an awl and a handheld reamer for increasing the size of the entry point in the bone. Then, the guide wire is introduced and confirmed radiologically.

**Figure 1:**
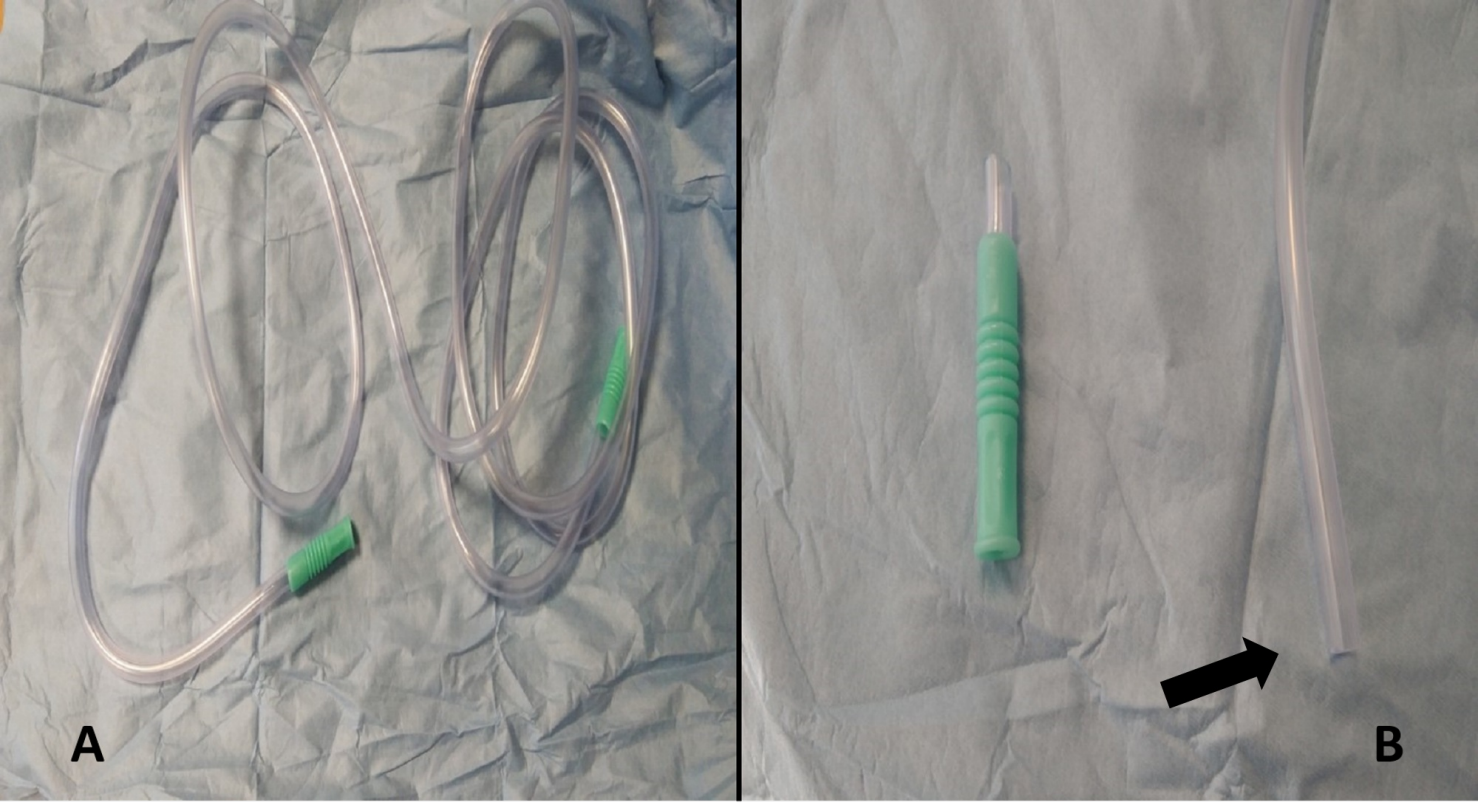
A) Suction tubing B) Tip of suction tubing after cut. A) Suction tubing (length - 3 m, internal diameter - 6.5 mm). B) The arrow indicating the tip of the suction tubing cut to make it compatible with the intramedullary canal.

When the guide wire is in position, we cut the tip of the suction tubing (length - 3 m; diameter - 6.5 mm). Figure 1 (A, B) shows the tubing.

The suction tube is inserted into the medullary canal through the entry point, sliding along the guide wire (which is usually 4 mm or 3.2 mm in diameter, depending on the manufacturer).

**Figure 2:**
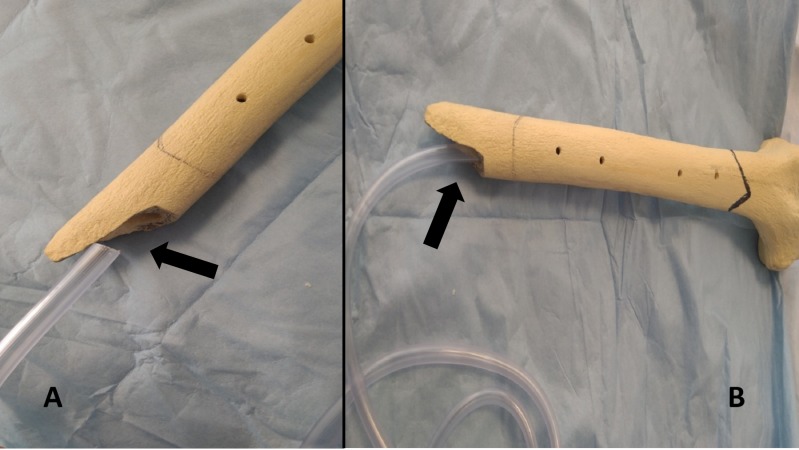
A) and B) In vitro suction tube introduced into the medullary canal of the femur model. A) and B) Arrows indicating the compatibility of the cut tip of the suction tubing with the femoral canal in vitro.

Figure 2 ( A, B) shows the in vitro representation of the tube insertion in the medullary canal.

We fully insert the suction tubing to the end of the IM bone and apply suction for two to three minutes. On average, 150 mL to 250 mL of material is suctioned out, depending on the size of the patient. After this, we continue with the rest of the procedure (including IM reaming). The process is repeated after reaming concludes as well.

**Figure 3:**
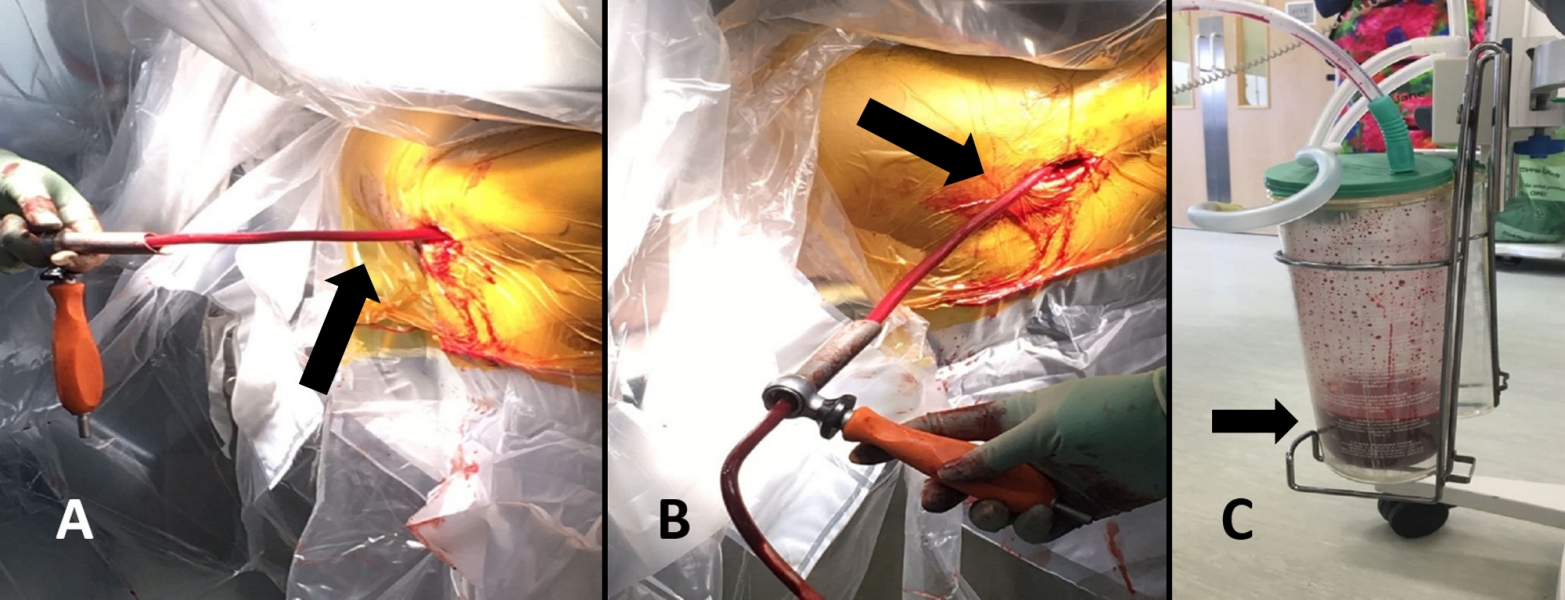
A) and B) The intraoperative setup. C) The suction container. A) and B) The arrows indicating the suction tubing inserted in the femoral canal through the entry point introduced on top of the guide wire (Guide wire insertion is the normal practice). C) The arrow points at the suction container, which has approximately 150 mL - 200 mL of medullary canal material removed during the procedure.

Figure 3 ( A, B, C) shows the intra-operative setup.

## Discussion

Fat embolism has been a widely reported complication of reaming that can develop into ARDS, which can be fatal [3]. FES is a triad of respiratory failure, petechial rash, and neurologic dysfunction. The etiology of this syndrome includes traumatic fractures, IM reaming and nailing, arthroplasties, pancreatitis, sickle cell disease, liposuction, and interosseous fluid administration, among others. Different mechanisms of this condition have been proposed, including the entry of bone marrow or fat droplets into the medullary canal or elevated IM pressure [3]. The compatibility of the suction tube with the intramedullary diameter (e.g., femur, tibia) is an important consideration. Sen et al. [4] highlighted this in their study that reported a mean femur medullary canal anteroposterior diameter of approximately 13 mm and a mean lateral diameter of 12 mm at the level of the diaphysis, which is the narrowest part of the long bones. The suction tubing we used has a uniform internal diameter of 6.5 mm.

In the literature, many anecdotal, experience-based methods and strategies have been described to minimize embolism. The goal is to ream but with a minimum increase in IM pressure (< 40 mm of Hg). Using sharp reamers with high rotation speed and avoiding high axial pressure and a high speed of advance has been recommended. The literature describes pharmacological or mechanical preventative measures. Pharmacological preventative options include prescribing prednisolone, heparin, or dextrose. The mechanical options include an inferior vena cava filter, which can decrease the embolic load and subsequently reduce risk [5]. There are a few customized reaming devices that work on the principle of flushing/suction being tested to keep the reaming head cool and to prevent a significant increase in IM pressure. However, those devices will be costly, and if we can achieve our goals using common equipment during an everyday procedure, both time and money will be saved.The improvisation we have described is cheap, effective, and can be done by anybody, anywhere.

## Conclusions

Our team has practiced this method for several years, with good results. This method is easy to use, very inexpensive, requires no new or additional equipment, and does not take long to setup or perform. We have encountered no unforeseen complications while using this technique. Given our success, we think it important that others consider the technique and assess it for areas where it may be improved.
